# BISRF-Net: A Baseline-Preserving Scale-Guided Residual Feature Routing Network for UAV-Based Inland Waterway Lock Monitoring

**DOI:** 10.3390/s26185777

**Published:** 2026-09-11

**Authors:** Boju Li, Xiaodong Lu, Sudong Xu, Haiyang Xu, Jiayi Deng

**Affiliations:** School of Transportation, Southeast University, Nanjing 211189, China

**Keywords:** UAV imaging, visual sensing, object detection, scale-sensitive detection, inland waterway monitoring

## Abstract

Unmanned aerial vehicle (UAV) acquired imagery provides a flexible non-contact visual sensing modality for monitoring inland waterway locks, yet reliable perception remains challenging due to significant scale variation among targets, as well as partial visibility, low contrast, water-surface texture variation, and complex backgrounds. To address these issues, this study proposes BISRF-Net, a baseline-preserving scale-guided residual feature routing network built upon the CBv2 Faster R-CNN framework. The method retains the original backbone, feature pyramid network, and region proposal network, while introducing scale-guided residual routing at the region-of-interest (ROI) refinement stage. Through identity-preserved residual addition, the baseline ROI representation is preserved and enhanced with complementary scale-sensitive information without altering the original feature pathway. Experiments conducted on the UAV image subset of the TROUT lock-monitoring dataset demonstrate that BISRF-Net maintains comparable overall detection performance while improving medium-scale target representation. Repeated trials with different random seeds further assess the robustness of the proposed method and indicate that its main benefit lies in medium-scale target refinement. Ablation and computational analyses further show that the proposed design enables targeted ROI-level refinement with additional computational cost. These findings highlight the potential of scale-guided ROI feature refinement for robust UAV-based visual sensing in complex inland waterway environments.

## 1. Introduction

Inland waterway transport is an important component of sustainable freight and regional mobility systems, particularly in regions where rivers, canals, and navigation locks support intensive cargo movement. Compared with road-based transport, inland navigation can reduce congestion, energy consumption, and emissions, but its operational safety depends on the reliable functioning of navigation infrastructure and the timely perception of vessels, lock gates, and surrounding facilities [1,2]. Navigation locks are especially critical nodes because vessel manoeuvring, infrastructure interaction, and traffic control occur within a confined spatial area. Recent advances in high-resolution remote sensing, particularly unmanned aerial vehicles (UAVs), provide a promising sensing platform for such environments, as UAVs can acquire detailed imagery from flexible viewpoints with relatively low deployment costs [3,4]. UAV platforms have also been widely used in photogrammetric and LiDAR mapping [5], hydrological and surface-water monitoring [6,7], and marine monitoring [8], indicating their broader potential for water-related remote sensing applications. In the context of inland waterway locks, UAV-based monitoring is not intended to replace existing lock-control systems or fixed surveillance cameras. Instead, it provides supplementary mobile visual sensing for temporary inspection, blind-area observation, emergency response, infrastructure inspection, and observing vessel–gate spatial relationships from flexible overhead or oblique viewpoints. Compared with fixed camera installations, UAVs can provide adjustable viewing angles and temporary coverage of areas that are difficult to observe from fixed infrastructure. However, UAV-based monitoring also introduces non-negligible challenges. Variations in flight altitude, camera angle, platform motion, wind disturbance, illumination, water-surface reflection, and limited flight endurance can affect image quality and target appearance [4,8]. More importantly, repeated down-sampling operations in deep detection networks may weaken or even suppress the already limited visual cues of small objects, making them difficult to distinguish from surrounding background structures [9,10]. These difficulties are further amplified in inland waterway and lock-monitoring scenes, where complex backgrounds may include buildings, trees, bank-side infrastructure, elongated vessels with variable orientations, narrow lock gates, and navigation-critical targets that often appear at small or medium scales [11,12]. In addition, practical monitoring systems usually require real-time or near-real-time inference on deployable platforms, which places further demands on the balance between detection accuracy, model compactness, and computational efficiency [13,14,15]. Therefore, visual perception in UAV-based inland waterway lock monitoring should be regarded not simply as a generic object detection task, but as a scale-sensitive remote sensing problem embedded in a safety-critical navigation environment.

Recent advances in deep learning have made visual perception increasingly feasible for inland waterway and maritime monitoring. In fluvial scenes, early studies demonstrated that Faster R-CNN, SSD, and YOLO-based detectors could identify vessels and navigation-related objects with promising accuracy [13]. This research direction has since expanded from single-source image detection to more application-oriented monitoring tasks, including Automatic Identification System (AIS) visual fusion for inland vessel tracking and traffic surveillance [16], real-time barge detection from existing traffic cameras [14], and UAV-based navigation mark detection for waterway safety management [17]. Related studies have also improved water-surface object detection using Faster R-CNN variants [18] and addressed inland vessel detection under complex riverbank backgrounds with lightweight YOLO-based architectures [11,12]. Together, these studies indicate that deep neural networks can support more automated and timely waterway monitoring. At the same time, most existing studies are still developed for specific targets, viewpoints, or sensing configurations, such as shore-based cameras, traffic cameras, AIS–visual fusion, or general vessel detection, which means that their task settings only partially match UAV-based lock monitoring. This limitation is also reflected in available datasets. SeaShips provides a large-scale benchmark for ship detection from coastline surveillance videos [19], while the Singapore Maritime Dataset supports visual and near-infrared maritime perception from on-shore and on-board viewpoints [20]. Maritime benchmarks have also been developed for deep-learning-based object detection in maritime environments [21]. MODS and MaSTr1325 further extend maritime perception research toward unmanned surface vehicle (USV)-oriented obstacle detection and semantic segmentation [22,23], and recent USV vessel benchmarks provide more fine-grained vessel categories for autonomous surface-vehicle perception [24]. Although these datasets have improved reproducibility and benchmarking in maritime computer vision, their viewpoints, object categories, and task definitions are not specifically designed for UAV-based lock scenes, where vessels, lock gates, buildings, trees, and bank-side infrastructure must be detected simultaneously from overhead or oblique aerial imagery. In this regard, the TROUT dataset is more closely aligned with the present study because it focuses on intelligent waterway traffic monitoring using UAV and LiDAR integration and includes six lock-related categories: Building, Tree, Lock_gate, Fully_loaded_cargo_ship, Fully_loaded_container_ship, and Unladen_cargo_ship [25].

This dataset-level relevance leads to a more specific technical question: how to detect objects reliably when their operational importance does not correspond to their visual scale. In UAV-based lock monitoring, scale variation is not merely a generic aerial-image problem, but directly relates to navigation safety. Drone and aerial benchmarks such as VisDrone and UAVDT have shown that UAV imagery often contains dense object distributions, occlusion, camera motion, and small targets [26,27]. The Dataset for Object deTection in Aerial images (DOTA) further highlights the large variation in object scale, orientation, and shape in aerial scenes [28], while Common Objects in Context (COCO) provides a widely used evaluation protocol for comparing detection performance across small, medium, and large objects [29]. In inland lock scenes, large objects such as buildings, trees, and large vessels often dominate the image and can strongly influence overall detection performance. By contrast, navigation-critical objects may have small or medium image-space bounding-box areas when their visible annotated regions are limited, for example when a vessel has just entered the monitored field of view, when only part of a vessel is visible near the image boundary, or when a lock gate occupies a narrow region in the UAV image. As a result, a detector with high overall average precision may still be inadequate if it fails to maintain sensitivity to medium-scale vessels and small navigation-related structures. This problem is further intensified by the inherent limitations of small-object detection, where targets contain fewer pixels, weaker semantic cues, and are more vulnerable to background interference and feature down-sampling [9,10,15]. Tiny-object detection studies further show that small bounding boxes are highly sensitive to localization deviation, making detection performance unstable when targets occupy only a limited number of pixels [30]. Therefore, improving UAV-based lock monitoring requires not only stronger feature representation, but also a more controlled strategy for introducing scale-aware information into the detection pipeline.

Existing object detectors provide several mechanisms for addressing scale variation, but their direct application to this setting is not straightforward. Faster R-CNN established the two-stage detection paradigm by introducing a Region Proposal Network (RPN) that shares convolutional features with the detection network and generates candidate regions for subsequent refinement [31]. The Feature Pyramid Network (FPN) then became a standard solution for multi-scale detection by combining high-level semantic features with higher-resolution feature maps [32]. Later architectures further strengthened cross-scale information propagation: PANet shortened the information path between low-level and high-level features [33], EfficientDet introduced weighted bidirectional feature fusion [34], and NAS-FPN explored automatically designed feature-pyramid structures [35]. Beyond feature-pyramid networks, other detector components are also relevant to this study. Residual learning provides a stable mechanism for deep feature transformation [36], while ROIAlign improves the spatial accuracy of ROI-level feature extraction [37]. CBNet/CBNetV2 strengthens backbone representation through composite connections [38], and Cascade R-CNN shows that progressive region refinement can improve localization quality [39]. In addition, MMDetection offers a modular implementation and benchmarking framework for developing and evaluating such detectors [40].

In complex remote-sensing scenes, reliable detection also depends on the separation of target responses from background interference. This issue has been extensively studied in hyperspectral anomaly detection, where weak or sparse targets are often embedded in complex backgrounds. Frequency-domain and representation-based methods such as DFBSNet and S3CRAD improve anomaly saliency by modelling target–background differences and reducing background interference [41,42]. Related contrast- and gradient-based methods further address background suppression from different perspectives: HELWC combines empirical mode decomposition with local weighted contrast to reduce spectral noise and suppress background responses [43], while HLSC-SSGF exploits local sub-block contrast and spatial–spectral gradient features to enhance low-contrast targets and suppress background edges [44]. More recently, TBCNet adopts a twin-branch collaborative design to decouple anomaly enhancement from background reconstruction, highlighting the importance of balancing target representation and background suppression [45]. Although these methods are developed for hyperspectral anomaly detection rather than UAV-based RGB object detection for lock monitoring, they provide useful methodological references for background-aware feature refinement under cluttered remote-sensing conditions. In UAV-based inland waterway lock scenes, vessels, lock gates, bank-side infrastructure, vegetation, and water-surface textures often coexist within the same image. Therefore, the detection challenge is not only related to multi-scale feature fusion, but also to how navigation-relevant target responses can be refined without amplifying complex background interference.

Yet, in a strong two-stage detector, introducing additional feature enhancement needs to be treated carefully. The RPN, feature assignment, and ROI refinement stages depend on a relatively stable feature distribution. Directly replacing or heavily modifying the feature pathway may disturb proposal generation and weaken the advantages of the original baseline. This concern is particularly relevant to UAV-based lock monitoring, where reliable large-object detection must be preserved while scale-sensitive and navigation-relevant target representations are refined. This motivates a baseline-preserving strategy in which the original proposal pathway is retained and scale-aware enhancement is introduced only before ROI refinement.

To address the above challenges, this paper proposes a Baseline-Preserving Scale-Guided Residual Feature Routing Network (BISRF-Net) for scale-sensitive object detection in UAV-based inland waterway lock monitoring. The method is developed on a CBv2 Faster R-CNN baseline and is designed to refine scale-sensitive ROI representations, with a particular focus on navigation-relevant medium-scale targets, without disturbing the original proposal-generation pathway. Unlike methods that directly replace the feature pathway, BISRF-Net preserves the original CBNetV2-CBFPN features for RPN proposal generation and introduces scale-guided residual feature routing only before ROI refinement. The main contributions of this work are summarised as follows:

1. A scale-sensitive detection framework for UAV-based inland waterway lock monitoring is proposed. The framework focuses on navigation-critical objects in lock scenes, including lock gates and vessels, where target scale varies substantially due to UAV viewpoint, object distance, and the spatial structure of the lock-monitoring area.
2. A baseline-preserving feature routing strategy is developed for strong two-stage detectors. Instead of replacing the original feature pathway, BISRF-Net retains the CBNetV2-CBFPN features used by the RPN. This design reduces the risk of disturbing proposal generation while allowing additional scale-aware information to be introduced before ROI-level refinement.
3. An identity-preserved residual formulation is introduced to retain the original CBNet/FPN feature response during proposal-scale-conditioned refinement. The identity path is not a learnable feature-alignment module; instead, it serves as a baseline-preserving skip connection that limits unnecessary feature-distribution shifts.

## 2. Materials and Methods

**Figure 1:**
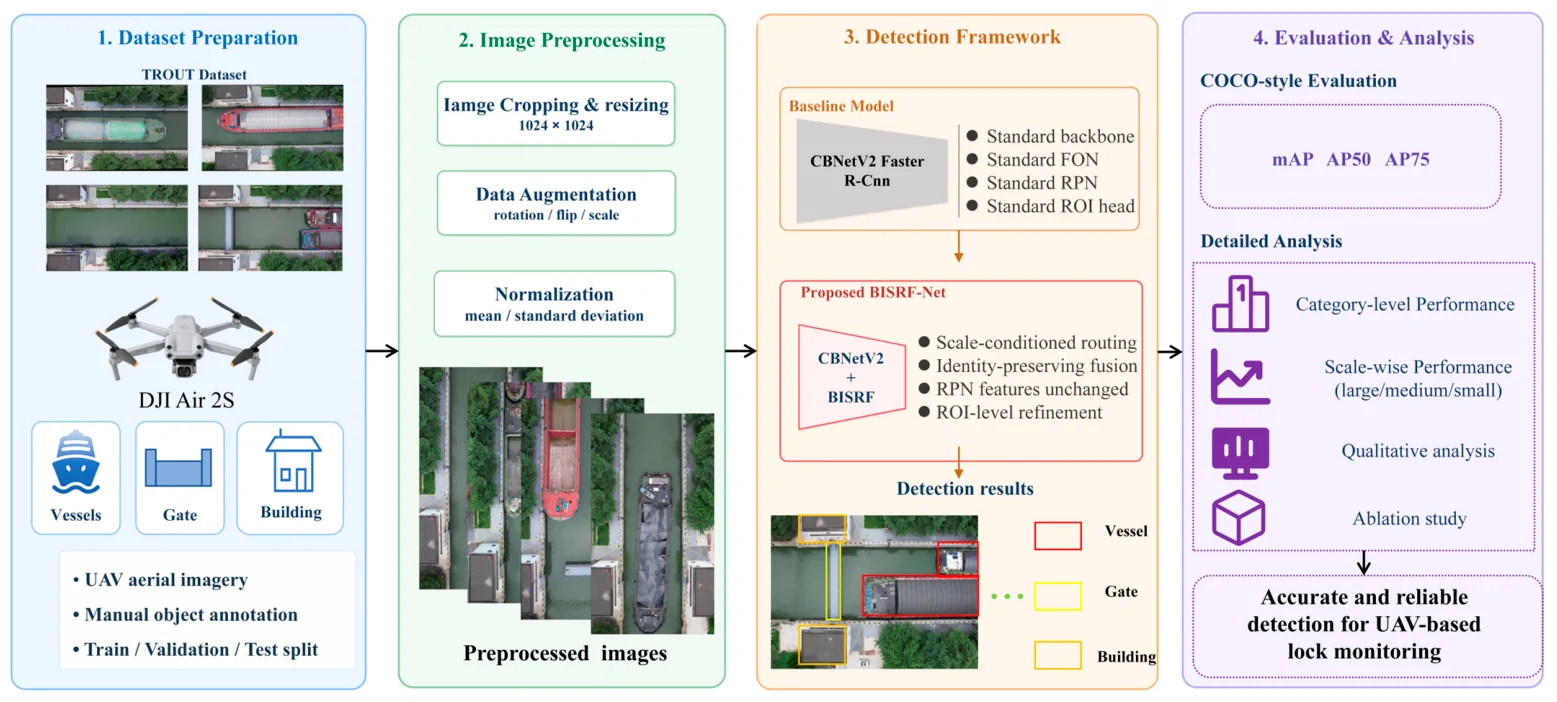
Overview of the methodological workflow adopted in this study.

An overview of the methodological workflow adopted in this study is presented in Figure 1. The workflow consists of four components: data preparation, image preprocessing, detection framework construction, and evaluation analysis. UAV images from the TROUT dataset are used as the experimental input and are processed through resizing, normalisation, and data augmentation. The object categories are organised into vessels, lock gates, and infrastructure-related objects, which together represent the main visual elements in inland waterway lock-monitoring scenes.

### 2.1. Dataset Description

This study uses the UAV image subset of the TROUT dataset for intelligent waterway traffic monitoring [25]. The public TROUT dataset release and usage instructions are available through the mmdetection_trout repository (https://github.com/serendipitylxd/mmdetection_trout, (accessed on 10 May 2026)). The imagery was acquired using a DJI Air 2S platform (SZ DJI Technology Co., Ltd., Shenzhen, China) in inland waterway lock environments, where vessels, lock gates, bank-side buildings, vegetation, and surrounding infrastructure coexist within the same field of view. In this study, only the UAV imagery is considered, and the task is formulated as two-dimensional object detection. Six object categories are included: Building, Tree, Lock_gate, Fully_loaded_cargo_ship, Fully_loaded_container_ship, and Unladen_cargo_ship. These categories represent both navigation-related targets and visually dominant background elements. In particular, lock gates and vessel categories are important for monitoring vessel movement and lock-area conditions, whereas buildings and trees contribute to the complex visual background of the monitored environment.

**Figure 2:**
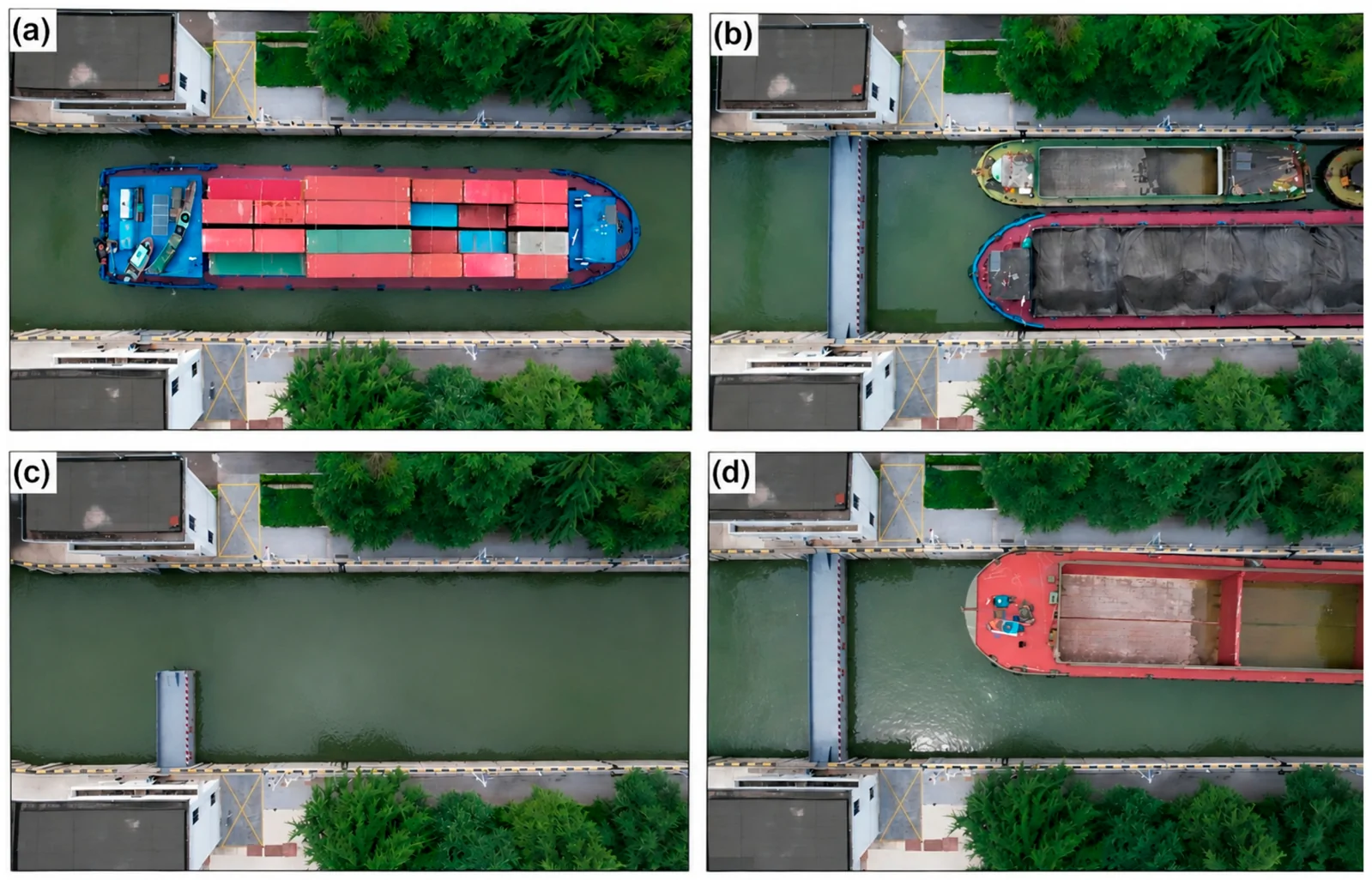
Example UAV images and annotated object categories in the TROUT dataset: (**a**) fully loaded container ship in the lock area; (**b**) vessel and bank-side background elements; (**c**) lock-gate region and surrounding infrastructure; and (**d**) vessel entering or passing through the monitored lock area.

The UAV imagery presents several challenges for reliable visual perception. Variations in aerial viewpoint, target distance, object orientation, boundary truncation, partial visibility, illumination, water-surface texture variation, and background complexity lead to substantial changes in apparent object scale and visual appearance. Water-surface texture variation, mild reflections, and visually cluttered bank-side regions may further weaken target boundaries and reduce the contrast between objects and their surroundings. These characteristics make the TROUT UAV subset suitable for evaluating scale-sensitive object detection in complex outdoor sensing environments. During model training and evaluation, all images were resized to 1024 × 1024 pixels to maintain a consistent input resolution across the compared detectors. Standard image normalisation was applied, and random horizontal flipping was used during training as data augmentation. No random augmentation was applied to the validation set. This unified preprocessing protocol was adopted to ensure that performance differences mainly reflect detector architecture and feature-routing design rather than inconsistencies in image resolution or evaluation settings. Table 1 summarises the object categories used in this study and their relevance to inland waterway lock monitoring. Example annotated UAV images from the TROUT dataset are shown in Figure 2. These examples illustrate the coexistence of navigation-related categories, including Lock_gate and vessel classes, and contextual background categories, including Building and Tree, within inland waterway lock-monitoring scenes.

### 2.2. Dataset Split and Preprocessing

The original training and validation split provided by the TROUT dataset was used in this study to maintain consistency with the dataset configuration and to avoid additional sampling bias. The UAV image subset was used for the object detection task, and all annotations were organised in COCO-style format and loaded through the MMDetection framework. The detection task contains six categories: Building, Tree, Lock_gate, Fully_loaded_cargo_ship, Fully_loaded_container_ship, and Unladen_cargo_ship.

During training and evaluation, all images were resized to 1024 × 1024 pixels to ensure a consistent input resolution across different detection models. Maintaining a fixed input resolution is particularly important for UAV imagery because object scale and background complexity can directly influence detection stability. Di et al. showed that enhanced feature fusion can improve UAV small-object representation under scale variation [46]. Liu et al. further emphasised that robust UAV small-object detection requires lightweight model design while maintaining stable performance in complex aerial scenes [47]. Standard image preprocessing was applied, including image normalisation using the ImageNet mean and standard deviation values. Data augmentation was limited to commonly used detection augmentations, including random horizontal flipping. The validation set was evaluated without random augmentation to ensure stable and reproducible performance assessment. This preprocessing strategy was adopted so that performance differences between the baseline and the proposed BISRF-Net could be attributed mainly to detector architecture and feature-routing design, rather than to inconsistent image resolution or data-processing settings.

Table 2 reports the dataset split and object-instance distribution used in this study. The validation set contains 2000 images and 10,634 annotated instances. The distribution shows that Building and Tree account for a large proportion of the annotated objects, while Lock_gate and vessel-related categories contain fewer instances. This imbalance reflects the visual characteristics of UAV-based lock-monitoring scenes, where large background objects frequently appear together with navigation-critical infrastructure and vessels.

Table 2 summarises the image- and instance-level composition of the TROUT dataset under the released split. The training split was used for model training, the validation split was used for controlled model comparison, repeated random-seed evaluation, ablation analysis, proposal recall analysis, and category-level diagnosis, and the complete test split was reserved for held-out test-set evaluation of the CBv2 Faster R-CNN baseline and BISRF-Net after the validation-set analyses.

The TROUT dataset exhibits a distinct category-level scale imbalance. Building and Tree appear exclusively as large-scale objects, while most vessel categories are also dominated by large-scale instances. In contrast, Lock_gate spans small, medium, and large scales, making it the most scale-variable infrastructure category in the dataset. This distinction is important because detection difficulty is affected not only by object size, but also by the interaction between image-space scale, category distribution, and complex lock-scene backgrounds. Real-time UAV small-object detection studies show that stronger scale-sensitive feature processing can improve detection robustness [48]. UAV-specific detector designs also indicate that small and scale-variable targets often require specialised feature adaptation [49]. Context-aware and edge-preserving feature fusion has further been shown to improve small-object detection under complex UAV backgrounds [50]. Therefore, the detection challenge in TROUT is better understood as category-level scale imbalance in a lock-monitoring scene, rather than conventional dense small-object detection.

### 2.3. Baseline Detector

The baseline detector used in this study is a CBv2 Faster R-CNN model implemented within the MMDetection framework. Faster R-CNN is a representative two-stage object detector that separates object detection into proposal generation and region-level refinement. In the first stage, the Region Proposal Network (RPN) generates candidate object proposals from the feature maps extracted by the backbone and feature pyramid. In the second stage, the ROI head further refines these proposals by performing object classification and bounding-box regression [31]. This two-stage structure is particularly well-suited for UAV-based lock-monitoring scenes, where targets such as lock gates and vessels exhibit significant scale variation, demanding both reliable proposal recall and precise region-level refinement.

**Figure 3:**
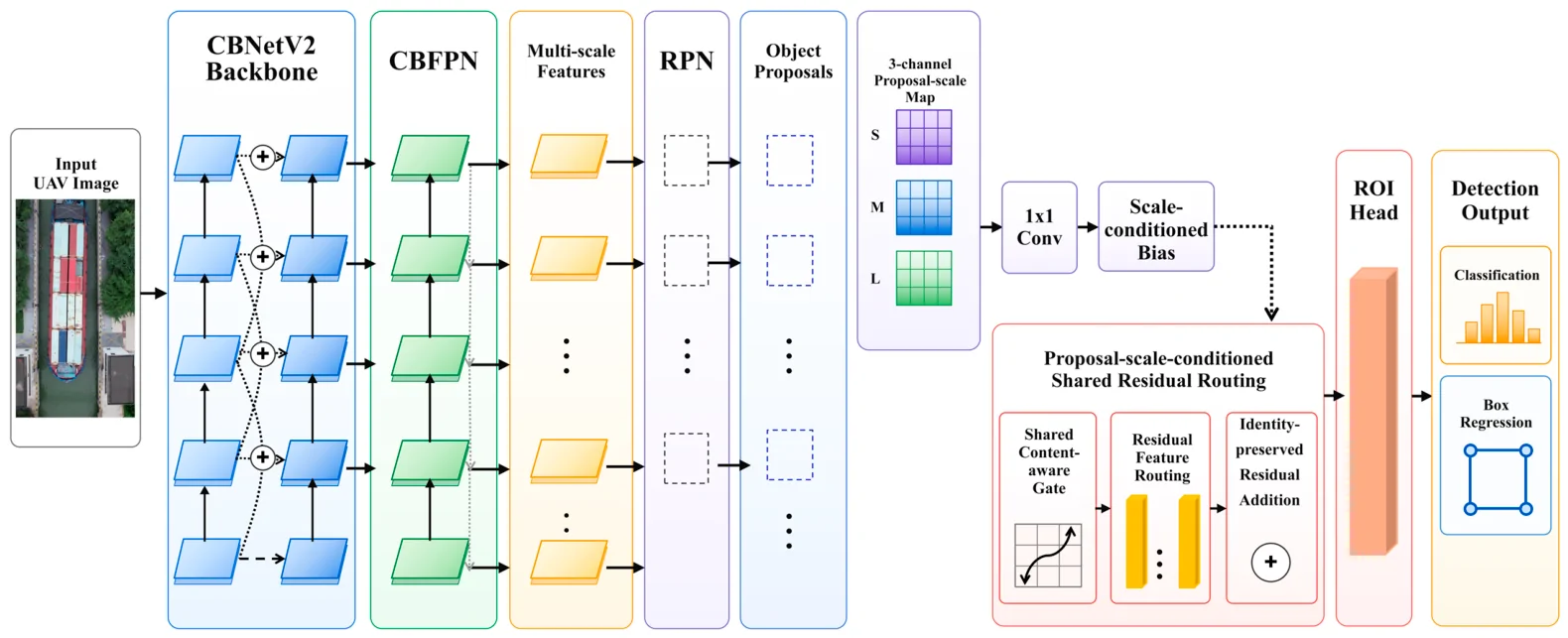
Architecture of the proposed BISRF-Net with baseline-preserving scale-guided residual feature routing.

To strengthen feature extraction, the baseline adopts a CBNetV2-based composite backbone and a CBFPN feature pyramid. Compared with a single-backbone detector, CBNetV2 improves representation capacity by connecting multiple backbone networks through composite connections, while the feature pyramid structure provides multi-scale feature maps for objects of different sizes [32,38]. This is important for the TROUT dataset because large objects such as buildings and trees coexist with navigation-critical targets, including lock gates and vessels that may appear at smaller or medium scales. Similar concerns have been reported in recent UAV detection studies, where scale variation and cluttered backgrounds require feature-fusion strategies that improve small-object representation without excessive computational cost [46]. Real-time UAV detection also requires a careful balance between accuracy and efficiency [48]. Lightweight UAV detection networks further reinforce the importance of reducing computational cost while maintaining robustness in small-object detection [51]. Therefore, the CBv2 Faster R-CNN baseline provides a strong reference model for evaluating scale-sensitive object detection in UAV-based inland waterway lock monitoring. The overall architecture of the proposed BISRF-Net is shown in Figure 3.

### 2.4. Baseline-Preserving Scale-Guided Residual Feature Routing Network

#### 2.4.1. Methodological Rationale

UAV-based inland waterway lock monitoring presents a distinctive detection challenge that differs from conventional small-object detection benchmarks. As characterised in Table 3, the TROUT dataset exhibits pronounced inter-class scale imbalance: background categories such as Building and Tree appear exclusively at large scale, whereas navigation-critical targets including Lock_gate and cargo vessels span multiple scale ranges depending on UAV altitude and viewing angle. This distribution indicates that the primary challenge is not dense small-object detection, but rather the simultaneous and reliable detection of targets across highly heterogeneous scale ranges within the same scene.

A naive approach to addressing this challenge would be to enhance or replace the feature extraction pathway prior to proposal generation. However, in a strong two-stage framework such as CBv2 Faster R-CNN, the Region Proposal Network (RPN) is tightly coupled with the feature distribution produced by the CBNetV2 backbone and CBFPN pyramid. Any modification introduced before the RPN risks perturbing the learned feature statistics, which may compromise proposal recall, particularly for medium-scale targets that rely on well-calibrated anchor matching. This consideration motivates a conservative post-proposal refinement strategy. In BISRF-Net, the feature maps used by the RPN are kept unchanged, and the proposed module is applied only before ROI-level classification and bounding-box regression. This design allows scale-sensitive ROI representations to be enhanced while maintaining the proposal-generation behaviour of the CBv2 Faster R-CNN baseline.

#### 2.4.2. Baseline-Preserving Proposal-Scale-Conditioned Feature Refinement

Let $x\in R^{\left(H\times W\times 3\right)}$ denote an input UAV image, and let $B\left(\cdot \right)$ and $N\left(\cdot \right)$ represent the CBNetV2 backbone and CBFPN neck, respectively. The baseline multi-scale feature maps are defined as

(1) $$F^{cb}=N\left(B\left(x\right)\right)={\left\{F_{i}^{cb}\right\}}_{i=2}^{5},$$

where $F^{cb}$ denotes the CBNet/FPN feature map at pyramid level *i*. In the proposed framework, these baseline feature maps are preserved for RPN proposal generation. This design keeps the feature distribution used by the RPN consistent with that of the original CBv2 Faster R-CNN baseline.

For each pyramid level *i*, the RPN predicts objectness scores and bounding-box offsets from the preserved feature map:

(2) $$\left(o_{i},t_{i}\right)=RPN_{i}\left(F_{i}^{cb}\right),$$

where $o_{i}\;$ denotes the objectness scores and $t_{i}$ denotes the bounding-box regression offsets at pyramid level i. Proposals from all pyramid levels are decoded and filtered by non-maximum suppression:

(3) $$P=NMS_{\tau_{nms}}\left({⋃}_{i=2}^{5}G\left(A_{i},o_{i},t_{i}\right)\right),$$

where $A_{i}$ denotes the anchor set at pyramid level i, $G\left(\cdot \right)$ represents the anchor decoding operation, and $NMS_{\tau_{nms}}$ denotes non-maximum suppression with threshold $\tau_{nms}$. The decoded proposals are therefore generated from the same upstream feature pathway as in the CBv2 Faster R-CNN baseline.

For each proposal $p_{j}\in P$, its visible image-space scale is computed as

(4) $$a_{i}=\sqrt{w_{i}h_{i}}$$

where $w_{i}$ and $h_{i}$ are the width and height of proposal $p_{j}$ after image resizing. Following the COCO-style scale thresholds, each proposal is assigned to one of three scale categories:

(5) $$g_{i}=\left\{\begin{array}{c} S,\;\;\;\\ M,\;\;\;\;\\ L,\;\;\;\; \end{array}\begin{array}{c} a_{i}<32\\ 32\leq a_{i}<96\\ a_{i}\geq 96 \end{array}\right.$$

where S, M, and L denote small-, medium-, and large-scale proposal groups, respectively. These scale labels are used to construct a three-channel proposal-scale map rather than to select independent scale-specific residual branches.

For each feature-pyramid level *i*, small-, medium-, and large-scale proposals are encoded as a three-channel proposal-scale map:

(6) $$M_{i}^{scale}\in R^{3\times H^{i}\times W^{i}}$$

where $H^{i}$ and $W^{i}$ denote the spatial height and width of the feature map at pyramid level *i*. The three channels represent the spatial occupancy of small-, medium-, and large-scale RPN proposals, respectively. The proposals used to construct this map are generated by the RPN during both training and inference, rather than from ground-truth boxes. Proposal scale is determined according to the COCO-style area thresholds in the resized input-image coordinates. Consistent with the FPN hierarchy, small proposals are mapped to P2–P3, medium proposals to P3–P5, and large proposals to P5–P6. Each proposal is projected from image coordinates to the corresponding feature-map coordinates and rasterized into its associated scale channel. When multiple proposals overlap, the map records scale-category occupancy rather than proposal density: same-scale overlaps are merged by binary union, while cross-scale overlaps are preserved as independent channel activations. Therefore, the proposal-scale map provides a compact spatial conditioning signal for proposal-scale-aware refinement without introducing proposal-count accumulation, confidence weighting, or hard competition among overlapping proposals.

Rather than employing three independent residual branches for different scale groups, BISRF-Net adopts a shared residual routing module. The three-channel proposal-scale map is first projected through a learnable 1×1 convolution to generate a scale-conditioned bias. This bias modulates the shared content-aware routing gate at each feature-pyramid level:

(7) $$G_{i}=\sigma \left(G_{\mathrm{shared}}\left(\left[F_{i}^{\mathrm{cb}},F_{i}^{\mathrm{aux}}\right]\right)+\beta_{i}C_{i}\left(M_{i}^{\mathrm{scale}}\right)\right)$$

where $F_{i}^{\mathrm{cb}}$ denotes the original CBNet/FPN feature map at pyramid level *i*, $F_{i}^{\mathrm{aux}}$ denotes the auxiliary CBFPN feature map at the same pyramid level, $\left[\cdot ,\cdot \right]$ denotes channel-wise concatenation, $G_{\mathrm{shared}}$ is the shared content-aware gate, $C_{i}\left(\cdot \right)$ is the learnable 1 × 1 convolution that converts the proposal-scale map into a scale-conditioned bias, $\beta_{i}$ is a learnable modulation coefficient, and σ(⋅) is the sigmoid activation function. The auxiliary feature is used as a complementary routing input, rather than as an independent backbone or a scale-specific residual branch.

The routed feature is then obtained by adaptively combining the original CBNet/FPN feature and the auxiliary feature:

(8) $$F_{i}^{\mathrm{route}}=G_{i}⊙F_{i}^{\mathrm{cb}}+\left(1-G_{i}\right)⊙F_{i}^{\mathrm{aux}}$$

where ⊙ denotes element-wise multiplication. Finally, the refined feature map is generated through identity-preserved residual addition.

(9) $$F_{i}^{\mathrm{out}}=F_{i}^{\mathrm{cb}}+\alpha_{i}\left(F_{i}^{\mathrm{route}}-F_{i}^{\mathrm{cb}}\right)$$

where $\alpha_{i}$ is a learnable residual coefficient. This formulation keeps the original CBNet/FPN feature as the reference path. If the residual coefficient or the routed difference is small, $F_{i}^{\mathrm{route}}$ naturally remains close to $F_{i}^{\mathrm{cb}}$, thereby preserving the baseline feature response.

For final detection, proposals are assigned to pyramid levels following the standard FPN rule:

(10) $$l_{j}=\mathrm{clip}\left(floor(l_{0}+{log}_{2}(\sqrt{w_{j}h_{j}}/224)),2,5\right)$$

where $l_{j}$ denotes the assigned pyramid level of proposal $p_{j}$, $l_{0}$ = 4 is the reference level, and clip(⋅,2,5) restricts the assigned level to the available pyramid levels. ROIAlign is then applied to the refined feature map at the assigned level:

(11) $$r_{j}=\mathrm{ROIAlign}\left(F_{l_{j}}^{\mathrm{out}},p_{j}\right)$$

where $r_{j}$ denotes the pooled ROI feature. The resulting ROI feature is then passed to the ROI head for final classification and bounding-box regression:

(12) $$Y=\{H\left(r_{i}^{enh}\right){\}}_{i=1}^{N}$$

where H(⋅) denotes the ROI head, and Y represents the final detection outputs.

Overall, BISRF-Net uses the proposal-scale map to modulate a shared content-aware routing gate, rather than assigning ROIs to separate scale-specific residual branches. The gated features are incorporated through identity-preserved residual addition and then passed to the ROI head for final prediction. In this way, the module performs post-proposal residual refinement while keeping the upstream proposal generator unchanged.

### 2.5. Evaluation Metrics

In this study, object scale refers to the visible image-space bounding-box area after resizing the UAV image to 1024 × 1024 pixels. It does not refer to the physical size of the real-world object. Following the COCO evaluation protocol, small objects are defined as instances with bounding-box area smaller than 32^2^ pixels, medium objects as instances with bounding-box area between 32^2^ and 96^2^ pixels, and large objects as instances with bounding-box area greater than or equal to 96^2^ pixels. Therefore, a physically large vessel may be evaluated as a medium-scale instance if its visible annotated region occupies a medium-sized area in the resized UAV image.

Detection performance was evaluated using the standard COCO-style average precision (AP) protocol implemented in MMDetection. The primary metric is AP, which is defined as the mean average precision computed across IoU thresholds from 0.50 to 0.95 with a step size of 0.05. Two threshold-specific metrics were also reported: ${AP}_{50}$, calculated at a fixed IoU threshold of 0.50, and ${AP}_{75}$, calculated at a stricter IoU threshold of 0.75. These metrics provide complementary views of detection quality, with ${AP}_{50}$ reflecting general localization accuracy and ${AP}_{75}$ placing greater emphasis on precise bounding-box regression. To assess scale-specific performance, ${AP}_{S}$, ${AP}_{M}$, and ${AP}_{L}$ were used following the COCO scale definitions, where ${AP}_{S}$ denotes AP for small objects with bounding-box area below $32^{2}$ pixels, ${AP}_{M}$ denotes AP for medium objects with area between $32^{2}$ and $96^{2}$ pixels, and ${AP}_{L}$ denotes AP for large objects with area greater than or equal to $96^{2}$ pixels. Given the scale distribution of the TROUT dataset, in which large-scale objects dominate while medium-scale instances include navigation-critical targets such as lock gates and vessels entering the monitoring area, ${AP}_{M}$ is particularly relevant to the proposed task. In addition to the standard COCO metrics summarized in Table 4, medium-scale vessel AP was reported separately for the three vessel categories to evaluate detection performance on navigation-critical targets. This metric provides a more targeted assessment of whether BISRF-Net improves vessel detection when vessels appear at medium scales before becoming visually dominant in UAV images.

## 3. Results

### 3.1. Overall Detection Performance Across Object Scales

Table 5 presents the scale-specific detection performance of BISRF-Net and the selected comparison models on the TROUT validation set. All models were evaluated using the same input resolution of 1024 × 1024 pixels and the COCO bbox AP protocol. The comparison includes representative one-stage detectors, transformer-based detectors, the CBv2 Faster R-CNN baseline, and the proposed BISRF-Net.

Among the compared models, ATSS R50 shows limited small-scale and medium-scale performance, with ${AP}_{S}$ of 0.293 and ${AP}_{M}$ of 0.590, although its ${AP}_{L}$ reaches 0.972. DINO R50 improves medium- and large-scale detection, achieving ${AP}_{M}$ of 0.764 and ${AP}_{L}$ of 0.997, but its ${AP}_{S}$ remains lower than that of the CBv2 Faster R-CNN baseline. Compared with the CBv2 Faster R-CNN baseline, BISRF-Net increases ${AP}_{M}$ from 0.770 to 0.802, corresponding to an absolute gain of 0.032, while maintaining ${AP}_{L}$ at 0.996. In the single-run comparison, ${AP}_{s}$ remains comparable to the baseline, decreasing only slightly from 0.634 to 0.632.

These results indicate that BISRF-Net achieves competitive medium-scale detection performance among the models evaluated under the same 1024 × 1024 input resolution. The improvement is concentrated in ${AP}_{M}$, rather than being uniformly distributed across all object scales. This behaviour suggests that the proposed ROI-level refinement is particularly effective for targets whose visual representations are sensitive to scale variation and background interference. This pattern is consistent with the design objective of BISRF-Net, which aims to enhance medium-scale ROI representations while preserving the stable large-object detection capability of the CBv2 Faster R-CNN baseline.

All experiments were conducted using MMDetection 3.3.0 with PyTorch 2.8.0 as the deep learning backend. All input images were resized to 1024 × 1024 pixels. Training was performed on an NVIDIA GeForce RTX 5070 GPU using the SGD optimiser.

To further isolate the effect of the proposed refinement module from the influence of backbone capacity, an additional backbone-controlled experiment was conducted using a standard Faster R-CNN R50-FPN detector. In this comparison, the baseline detector and the BISRF-enhanced detector share the same backbone, feature pyramid, RPN, training schedule, input resolution, and COCO-style evaluation protocol. The only architectural difference is the introduction of the proposal-scale-conditioned BISRF module.

As shown in Table 6, adding the BISRF module to the same Faster R-CNN R50-FPN detector increases AP from 0.9865 to 0.9874 and ${AP}_{M}$ from 0.7947 to 0.8054. Medium-scale vessel AP also increases from 0.7380 to 0.7521. These results suggest that the medium-scale improvement isnot solely a consequence of using the stronger CBNetV2 backbone in the main model, but can also be observed under a standard R50-FPN two-stage detector. At the same time, ${AP}_{S}$ decreases from 0.6192 to 0.5396, indicating that the proposed module should be interpreted as a targeted medium-scale refinement strategy rather than a general all-scale enhancement method.

To assess the stability of the observed performance pattern, both the CBv2 Faster R-CNN baseline and BISRF-Net were repeated three times with different random seeds, as summarized in Table 7.

The repeated random-seed evaluation further confirms this performance pattern. Across three independent trials, BISRF-Net maintains comparable overall AP to the CBv2 Faster R-CNN baseline. Its main advantage appears in medium-scale detection, where BISRF-Net achieves higher ${AP}_{M}$ than the baseline. In contrast, the improvement is not uniform across all object scales. These results further support the interpretation that BISRF-Net mainly provides targeted medium-scale feature refinement while preserving the strong large-object performance of the baseline detector.

After the validation-set analyses, the CBv2 Faster R-CNN baseline and BISRF-Net were further evaluated on the TROUT test split to assess their performance on the held-out test data. As reported in Table 8, BISRF-Net achieves an AP of 0.9852, compared with 0.9836 for the CBv2 Faster R-CNN baseline. The overall improvement is modest, but the scale-specific results show a clearer gain for medium-scale objects, with ${AP}_{M}$ increasing from 0.8189 to 0.8448. Medium-scale vessel AP also increases from 0.7729 to 0.8069, indicating improved recognition of navigation-relevant vessels at intermediate image-space scales. Meanwhile, ${AP}_{L}$ remains comparable between the two models, suggesting that the proposed refinement module does not noticeably compromise large-object detection on the test split. These results are consistent with the validation-set findings and further support the interpretation that BISRF-Net mainly provides targeted medium-scale feature refinement rather than uniform all-scale enhancement.

### 3.2. Per-Category Detection Analysis

Category-level results reveal a consistent pattern: BISRF-Net preserves baseline performance on dominant background categories while providing targeted improvements for navigation-critical targets. As shown in Table 9, AP values for Building and Tree remain at 0.99 for both models, confirming that the proposed proposal-scale-conditioned residual refinement does not affect the recognition of large visually prominent objects. Among navigation-critical categories, the most notable gain is observed for Unladen_cargo_ship (+0.04), followed by the two fully loaded vessel categories (+0.02 each) and Lock_gate (+0.01).

To evaluate vessel detection more directly, medium-scale vessel AP was computed separately for the three vessel categories. As shown in Table 10, BISRF-Net achieves a medium-scale vessel AP of 0.7483, compared with 0.7038 for the CBv2 Faster R-CNN baseline, corresponding to an absolute gain of 0.0445. Since vessels entering the monitored lock area frequently appear at medium scales before occupying a larger image region, medium-scale vessel AP provides a more task-relevant performance indicator than aggregate AP for this monitoring scenario. Together with the scale-specific results in Section 3.1, these findings indicate that BISRF-Net enhances the representation of medium-scale targets that are particularly sensitive to viewpoint variation, partial visibility, boundary interference, and complex bank-side backgrounds, while preserving the strong large-object performance of the CBv2 Faster R-CNN baseline.

### 3.3. Ablation Analysis of the Baseline-Preserving Residual Routing Strategy

To examine the contribution of each component in BISRF-Net, an ablation analysis was conducted on the TROUT validation set. The ablation design focuses on four key factors of the proposed framework: the proposal-scale map, the scale-conditioned bias, the shared routing gate, and the identity-preserved residual connection. The proposal-scale map and scale-conditioned bias are used to introduce explicit scale information, the shared gate performs content-aware feature routing, and the identity residual connection preserves the original CBNet/FPN feature response during refinement. The results are summarised in Table 11.

The ablation results in Table 11 illustrate the effect of different feature-routing designs on scale-specific detection performance. The CBv2 Faster R-CNN baseline obtains ${AP}_{s}$, ${AP}_{M}$, and ${AP}_{L}$ values of 0.634, 0.770, and 0.996, respectively, providing a strong reference for large-object detection but leaving room for improvement in medium-scale vessel-related targets.

Direct feature replacement increases ${AP}_{M}$ to 0.806, which is the highest ${AP}_{M}$ among the ablation variants. However, this gain is accompanied by a substantial decrease in ${AP}_{s}$ from 0.634 to 0.238 and a slight decrease in ${AP}_{L}$ from 0.996 to 0.993. This trade-off suggests that directly replacing the original feature representation can enhance medium-scale responses, but it may also disturb the feature distribution learned by the baseline detector and lead to unstable performance across object scales.

The residual routing variant without identity preservation achieves ${AP}_{M}$ of 0.803 and medium-scale vessel AP of 0.7245. Compared with direct feature replacement, this variant alleviates the degradation of ${AP}_{s}$, but its small-object performance remains lower than that of BISRF-Net. This result shows that residual routing alone is not sufficient to fully preserve the baseline feature response during refinement.

The standard residual routing variant without scale cue further clarifies the role of explicit proposal-scale information. Although this variant retains the shared gate and identity residual connection, removing the proposal-scale map and scale-conditioned bias leads to only limited improvement over the baseline, with ${AP}_{M}$ increasing from 0.770 to 0.775 and medium-scale vessel AP increasing from 0.7038 to 0.7086. In contrast, BISRF-Net achieves ${AP}_{M}$ of 0.802 and the highest medium-scale vessel AP of 0.7483 after incorporating proposal-scale-conditioned modulation.

Overall, BISRF-Net provides a more balanced refinement effect than the alternative variants. Although direct feature replacement produces a slightly higher ${AP}_{M}$, it severely degrades ${AP}_{s}$ and does not achieve the best medium-scale vessel AP. BISRF-Net preserves ${AP}_{L}$ at 0.996, keeps ${AP}_{s}$ close to the baseline, and provides the strongest improvement for medium-scale vessel detection. These results demonstrate that proposal-scale-conditioned residual refinement is more suitable than direct feature replacement for improving task-relevant medium-scale detection while maintaining the stability of the original CBv2 Faster R-CNN feature representation.

RPN proposal recall was measured to characterise the proposal-generation behaviour of different feature-routing designs. The evaluation followed the COCO proposal evaluation protocol on the TROUT validation set. Average recall was computed over IoU thresholds from 0.50 to 0.95 with maximum proposal numbers of 100, 300, and 1000. The resulting overall and scale-specific proposal recall values are summarized in Table 12, with scale-specific proposal recall reported for small-, medium-, and large-scale objects under 1000 proposals per image.

The proposal recall results show that the original CBv2 Faster R-CNN baseline already provides highly reliable proposal coverage on the TROUT validation set, with an ${AR}_{1000}$ of 0.993. Direct feature replacement reduces ${AR}_{1000}$ from 0.993 to 0.984 and decreases ${AR}_{M}$ from 0.900 to 0.874. This decline suggests that directly replacing the original feature pathway may disturb the proposal-generation process, especially for medium-scale objects.

The residual routing variant without identity preservation maintains a high overall ${AR}_{1000}$ of 0.992, but its ${AR}_{s}$ decreases to 0.642. BISRF-Net obtains an ${AR}_{1000}$ of 0.989, which remains close to the baseline, while ${AR}_{M}@1000$ is maintained at 0.901 and ${AR}_{S}$ increases to 0.705. This pattern indicates that the proposed module introduces post-proposal refinement without substantially degrading proposal recall.

To further evaluate the computational cost introduced by the proposed module, the parameter count, FLOPs, and GPU memory consumption of the CBv2 Faster R-CNN baseline and BISRF-Net are reported in Table 13. Both models were evaluated using the same input size of 1024 × 1024. Compared with the baseline, BISRF-Net increases the number of parameters from 69.25 M to 83.62 M and the FLOPs from 0.289 T to 0.391 T. GPU memory consumption also increases from 876 MB to 967 MB. These results indicate that the proposal-scale-conditioned residual routing module introduces additional computational complexity. Therefore, BISRF-Net improves medium-scale detection at the cost of increased model complexity, and the current version is more suitable for accuracy-oriented UAV-based lock monitoring rather than strict lightweight real-time deployment.

### 3.4. Qualitative Assessment in UAV-Based Lock-Monitoring Scenes

**Figure 4:**
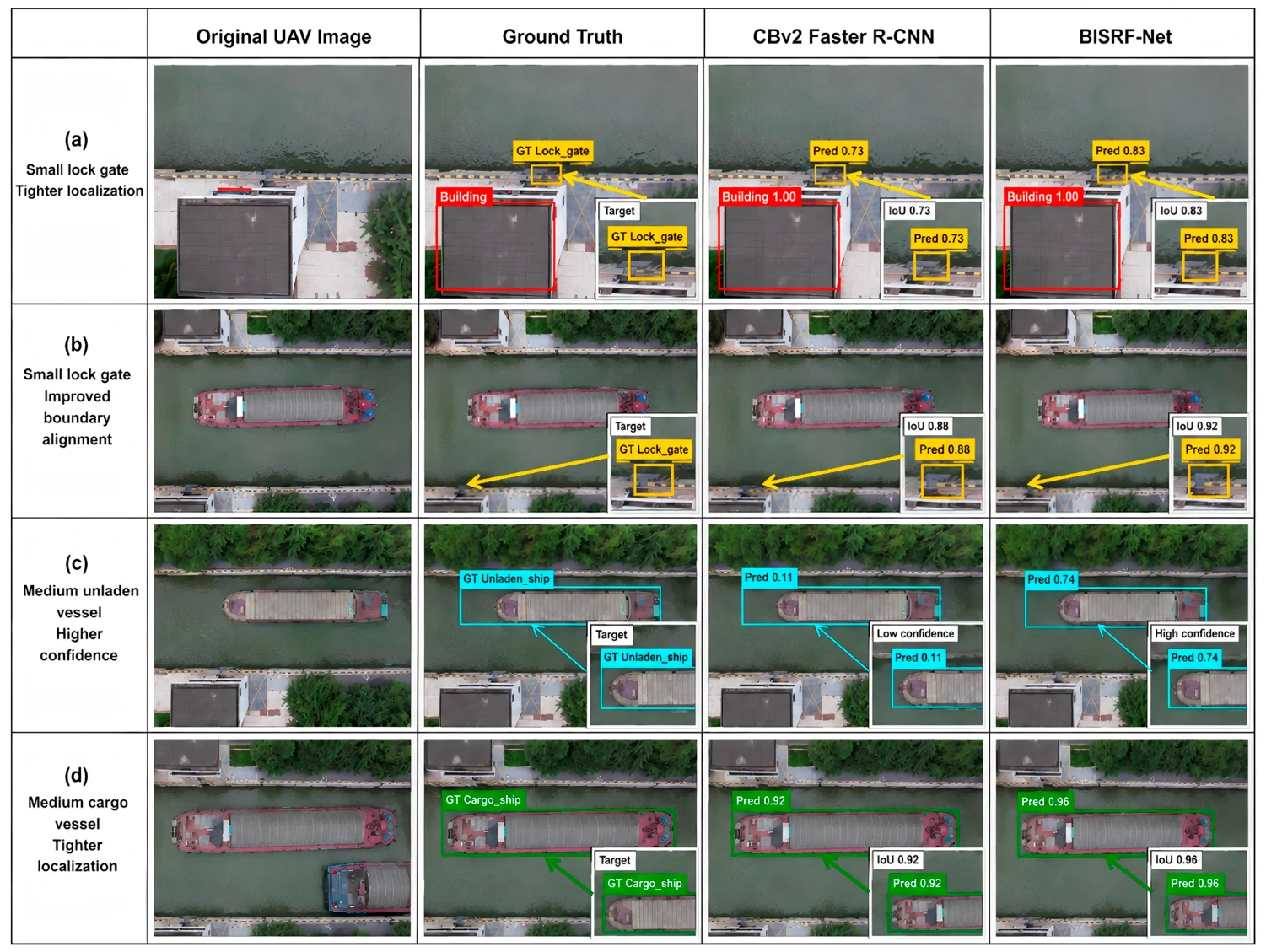
Qualitative comparison of detection results in UAV-based inland waterway lock-monitoring scenes.

Representative qualitative detection results are shown in Figure 4 for the CBv2 Faster R-CNN baseline and the proposed BISRF-Net in UAV-based inland waterway lock-monitoring scenes. The selected examples cover typical visual conditions in the TROUT dataset, including vessels entering the monitoring area, lock-gate regions, large background infrastructure, and scenes with complex bank-side objects. These examples provide visual confirmation of the quantitative improvements reported in Section 3.1, Section 3.2 and Section 3.3, including enhanced medium-scale target detection and preserved large-object recognition under complex UAV viewing conditions.

Compared with the baseline, BISRF-Net produces more reliable detections for medium-scale targets in representative UAV-based lock-monitoring scenes. In scenes where vessels are entering the monitored lock area, the proposed model provides more complete vessel localization and reduces missed detections. For lock-gate regions, BISRF-Net also shows improved sensitivity to narrow infrastructure objects, which is consistent with the category-level improvement observed for Lock_gate. At the same time, large background categories such as buildings and trees remain reliably detected, indicating that the ROI-level enhancement does not visibly weaken the baseline’s response to dominant large-scale objects.

## 4. Discussion

### 4.1. Medium-Scale Perception Under UAV Imaging Conditions

The experimental results show that BISRF-Net mainly benefits medium-scale detection under UAV-based lock-monitoring conditions. Compared with the CBv2 Faster R-CNN baseline, ${AP}_{M}$ increases from 0.770 to 0.802, while ${AP}_{S}$ remains close to the baseline and ${AP}_{L}$ is preserved at 0.996. This scale-wise pattern is consistent with the visual composition of the TROUT set, which is dominated by large background objects and large vessel instances, but still contains navigation-relevant targets such as vessels entering the monitored area and narrow lock-gate structures at medium image-space scales.

The improvement in ${AP}_{M}$ indicates that medium-scale targets are the most responsive to the proposed proposal-scale-conditioned residual refinement. These targets are not as visually dominant as large buildings, trees, or fully visible vessels, but they still preserve sufficient spatial structure for feature refinement. This explains why BISRF-Net shows clearer gains in medium-scale detection than in very small-object detection. Medium-scale vessel AP also increases from 0.7038 to 0.7483, suggesting that the proposed method improves vessel-related perception at a task-relevant scale for UAV-based lock monitoring. In practical lock-operation scenarios, vessels may first appear as medium-scale annotated instances before becoming large and visually dominant in the image. Improving detection at this stage may support earlier visual perception of vessel movement, although the present study evaluates object detection only and does not directly assess downstream navigation decisions.

### 4.2. Role of Baseline-Preserving Residual Routing

The ablation results clarify why residual post-proposal refinement is preferable to direct feature replacement in this setting. Direct feature replacement increases ${AP}_{M}$, but it causes a substantial decrease in ${AP}_{S}$ and a slight decrease in ${AP}_{L}$. Residual routing without identity preservation also improves medium-scale performance, but its ${AP}_{S}$ and ${AP}_{L}$ are lower than those of the full BISRF-Net. These results indicate that higher medium-scale AP alone does not necessarily imply a better detector for UAV-based lock monitoring. Since the scene contains both dominant large objects and navigation-relevant medium-scale targets, the detector should improve medium-scale perception without sacrificing stable recognition of other object scales.

The identity-preserved residual formulation contributes to this balance by using the baseline feature response as a stable reference and introducing a complementary residual component conditioned on proposal-scale information. This controlled refinement allows BISRF-Net to improve ${AP}_{M}$ while avoiding severe degradation in ${AP}_{S}$ and ${AP}_{L}$. The results therefore suggest that, in UAV-based lock-monitoring scenes, proposal-scale information is more effective when introduced as residual modulation rather than as direct feature replacement.

### 4.3. Scale-Wise Stability and Implications for UAV Visual Sensing

The results also show that BISRF-Net preserves the recognition of large-scale objects. ${AP}_{L}$ remains at 0.996, and the category-level AP values for Building and Tree remain unchanged at 0.99. This is important because large infrastructure and vegetation categories provide scene context in UAV-based lock monitoring. A detector that improves vessel or lock-gate detection but weakens the recognition of large background structures would be less reliable for operational monitoring, where surrounding infrastructure, bank-side objects, and vessel positions must be interpreted together.

The comparison with other detectors evaluated at the same 1024 × 1024 input resolution further places BISRF-Net in a broader detection context. GFL achieves strong overall performance but shows lower ${AP}_{S}$ and ${AP}_{M}$ than BISRF-Net, whereas TOOD achieves the highest overall AP and matches BISRF-Net in ${AP}_{M}$. Nevertheless, BISRF-Net obtains a slightly higher medium-scale vessel AP than TOOD, indicating that its advantage is task-specific rather than universal. These results suggest that aggregate AP alone may not fully characterise scale-sensitive perception performance in UAV-based lock-monitoring scenes.

From a visual sensing perspective, the results indicate that downstream perception performance is strongly influenced by the effective image scale of targets rather than by semantic category alone. Objects observed from UAV platforms can undergo substantial apparent-scale changes because of variations in viewpoint, distance, and scene geometry. The proposed baseline-preserving strategy addresses this issue at the region-refinement stage by introducing scale-conditioned residual information without modifying the upstream proposal-generation pathway. This separation is useful in UAV-based lock-monitoring scenes where the original detector already provides reliable large-scene perception but remains sensitive to scale-dependent feature degradation for medium-scale targets.

From an application perspective, this balance is more meaningful than maximising aggregate AP alone. In inland waterway lock monitoring, the timely detection of vessels entering the monitored area, the recognition of lock gates, and the preservation of large-scene context are all important. The qualitative results show that BISRF-Net can produce higher-confidence vessel detections and tighter lock-gate localization in representative cases. These improvements may support earlier visual perception of vessels entering the monitored area and better interpretation of navigation-relevant objects in UAV imagery.

### 4.4. Limitations and Future Work

Several limitations remain. First, the improvement in small-object detection is limited. Although ${AP}_{s}$ remains close to the baseline in the single-run comparison, the repeated experiments show a decrease from 0.634 ± 0.010 to 0.620 ± 0.010. Therefore, the present results do not provide sufficient evidence to claim that BISRF-Net improves very small target detection. This limitation may be related to the limited number of small instances in the TROUT validation set and the fact that very small targets contain fewer pixels and are more sensitive to feature down-sampling, localization deviation, and background interference.

Second, this study uses only the UAV image subset of the TROUT dataset. Although TROUT provides multi-modal observations, the LiDAR modality is not incorporated into the present framework. Future research could explore UAV–LiDAR fusion to improve geometric understanding of vessel boundaries, lock gates, and bank-side infrastructure. This may be particularly useful for cases involving partial visibility, water reflection, or ambiguous object boundaries. If more UAV-based lock-monitoring data under complex illumination, weather, reflection, or low-visibility conditions become available in the future, further experiments will be conducted to evaluate the robustness and generalisation ability of BISRF-Net under more challenging environmental conditions.

Third, although this study has added a preliminary computational complexity analysis, the deployment efficiency of BISRF-Net still requires further optimisation. The current evaluation reports parameter count, FLOPs, and GPU memory consumption, showing that the proposed scale-guided residual routing module introduces additional computational cost. Therefore, the present version of BISRF-Net is more suitable for accuracy-oriented UAV-based lock-monitoring scenarios rather than strict real-time deployment on lightweight edge devices. Since practical waterway monitoring may require real-time or near-real-time inference, future work will focus on lightweight routing design, inference acceleration, and deployment evaluation under operational hardware constraints. Further experiments under different UAV altitudes, camera angles, weather conditions, and illumination settings are also needed to evaluate the robustness of BISRF-Net beyond the current TROUT evaluation setting.

In addition, this study focuses on object detection rather than downstream decision-making. In practical navigation monitoring, detection outputs are only one part of a larger perception system. Future work could integrate BISRF-Net with multi-frame tracking, vessel trajectory analysis, lock-entry event recognition, and navigation risk assessment. Such extensions would help transform scale-sensitive object detection into a more complete intelligent monitoring framework for inland waterway lock operations.

## 5. Conclusions

This study proposes BISRF-Net, a baseline-preserving residual feature routing network for scale-sensitive visual perception in UAV-based inland waterway lock-monitoring scenarios. The method preserves the CBNetV2-CBFPN backbone and RPN proposal-generation pathway, while introducing proposal-scale-conditioned residual refinement to improve medium-scale feature representation. This design aims to enhance navigation-relevant targets such as vessels and lock gates without directly replacing the original feature pathway of the baseline detector.

Experiments on the UAV image subset of the TROUT dataset show that BISRF-Net maintains comparable overall AP and preserves strong large-object performance, while providing targeted improvements for medium-scale detection. The main benefit is reflected in ${AP}_{M}$ and medium-scale vessel AP rather than uniform improvement across all object scales. Repeated random-seed evaluation and ablation analysis further indicate that small-object detection does not improve consistently, and that direct feature replacement can lead to unstable scale-wise performance.

Overall, the results suggest that proposal-scale-conditioned residual refinement is a useful strategy for medium-scale target perception in UAV-based lock-monitoring imagery. Although the added held-out test-set evaluation provides further confirmation on the TROUT dataset, broader cross-dataset and multi-scene validation is still required. Future work will focus on lightweight routing design, real-time deployment evaluation, robustness under more diverse UAV imaging conditions, and integration with multi-modal information such as LiDAR.

## Figures and Tables

**Table 1:** Object categories in the TROUT dataset.

Category	Type	Relevance to Lock Monitoring
Building	Background infrastructure	Bank-side visual interference
Tree	Background object	Complex bank-side background
Lock_gate	Navigation infrastructure	Critical lock operation object
Fully_loaded_cargo_ship	Vessel	Navigation-critical target
Fully_loaded_container_ship	Vessel	Navigation-critical target
Unladen_cargo_ship	Vessel	Navigation-critical target

**Table 2:** Dataset split and object-instance distribution.

Split	Images	Instances	Building	Tree	Lock_Gate	Fully_Loaded_Cargo_Ship	Fully_Loaded_Container_Ship	Unladen_Cargo_Ship
Training	6000	31,963	12,000	12,000	1377	5516	411	659
Validation	2000	10,634	4000	4000	454	1809	133	238
Test	8000	42,620	16,000	16,000	1829	7345	548	898

**Table 3:** Scale distribution of object categories in the TROUT validation set.

Category	Small (%)	Medium (%)	Large (%)
Lock_gate	3.5	28.9	67.6
Fully_loaded_cargo_ship	0.2	3.8	96.0
Fully_loaded_container_ship	0.0	4.5	95.5
Unladen_cargo_ship	0.0	2.1	97.9
Building	0.0	0.0	100.0
Tree	0.0	0.0	100.0

**Table 4:** Evaluation metrics used in this study.

Metric	Definition
AP	Mean AP across IoU thresholds from 0.50 to 0.95
${AP}_{50}$	AP at IoU = 0.50
${AP}_{75}$	AP at IoU = 0.75
${AP}_{S}$	AP for small objects with area < $32^{2}$ pixels
${AP}_{M}$	AP for medium objects with $32^{2}\leq$ area < $96^{2}$ pixels
${AP}_{L}$	AP for large objects with area $\geq 96^{2}$ pixels

**Table 5:** Overall and scale-specific detection performance on the TROUT validation set.

Model	AP	${AP}_{50}$	${AP}_{75}$	${AP}_{S}$	${AP}_{M}$	${AP}_{L}$
Deformable DETR R50	0.814	0.968	0.920	0.038	0.340	0.836
DAB-DETR R50	0.947	0.994	0.986	0.293	0.593	0.963
ATSS R50	0.962	0.992	0.988	0.293	0.590	0.972
YOLOX-S	0.964	0.990	0.986	0.370	0.556	0.978
YOLOX-T	0.974	0.992	0.990	0.537	0.727	0.986
GFL R50	0.987	0.993	0.990	0.419	0.724	0.997
DDQ-DETR R50	0.986	0.995	0.993	0.552	0.770	0.996
DINO R50	0.986	0.993	0.990	0.479	0.764	0.997
TOOD R50	0.989	0.996	0.993	0.535	0.802	0.998
CBv2 Faster R-CNN baseline	0.987	0.995	0.992	0.634	0.770	0.996
BISRF-Net	0.986	0.997	0.993	0.632	0.802	0.996

**Table 6:** Backbone-controlled comparison under the same R50-FPN detector.

Model	Backbone	BISRF Module	AP	${AP}_{50}$	${AP}_{75}$	${AP}_{S}$	${AP}_{M}$	${AP}_{L}$	Medium-Scale Vessel AP
Faster R-CNN	R50-FPN	No	0.9865	0.9966	0.9933	0.6192	0.7947	0.9960	0.7380
Faster R-CNN + BISRF	R50-FPN	Yes	0.9874	0.9965	0.9932	0.5396	0.8054	0.9964	0.7521

**Table 7:** Robustness comparison under different random seeds.

Model	AP	${AP}_{50}$	${AP}_{75}$	${AP}_{S}$	${AP}_{M}$	${AP}_{L}$
CBv2 Faster R-CNN	0.987 ± 0.000	0.996 ± 0.001	0.992 ± 0.000	0.634 ± 0.010	0.793 ± 0.007	0.996 ± 0.000
BISRF-Net	0.986 ± 0.000	0.996 ± 0.001	0.993 ± 0.000	0.620 ± 0.009	0.802 ± 0.008	0.996 ± 0.000

**Table 8:** Test-set evaluation on the TROUT test split.

Model	AP	${AP}_{S}$	${AP}_{M}$	${AP}_{L}$	Medium-Scale Vessel AP
CBv2 Faster R-CNN	0.9836	0.2372	0.8189	0.9930	0.7729
BISRF-Net	0.9852	0.2437	0.8448	0.9938	0.8069

**Table 9:** Category-level AP comparison on the TROUT validation set.

Category	CBv2 Faster R-CNN	BISRF-Net	Difference
Building	0.99	0.99	0.00
Tree	0.99	0.99	0.00
Lock_gate	0.95	0.96	+0.01
Fully_loaded_cargo_ship	0.85	0.87	+0.02
Fully_loaded_container_ship	0.80	0.82	+0.02
Unladen_cargo_ship	0.70	0.74	+0.04

**Table 10:** Medium-scale vessel detection performance on the TROUT validation set.

Model	Medium-Scale Vessel AP
TOOD R50	0.7433
CBv2 Faster R-CNN	0.7038
BISRF-Net	0.7483

**Table 11:** Ablation analysis of the proposed BISRF-Net components on the TROUT validation set.

Model	Proposal-Scale Map	Scale-Conditioned Bias	Shared Gate	Identity Residual	${AP}_{S}$	${AP}_{M}$	${AP}_{L}$	Medium-Scale Vessel AP
CBv2 Faster R-CNN baseline	No	No	No	No	0.634	0.770	0.996	0.7038
Direct feature replacement	Yes	Yes	Yes	No	0.238	0.806	0.993	0.7321
Residual routing without identity preservation	Yes	Yes	Yes	No	0.568	0.803	0.995	0.7245
Standard residual routing without scale cue	No	No	Yes	Yes	0.542	0.775	0.995	0.7086
BISRF-Net	Yes	Yes	Yes	Yes	0.632	0.802	0.996	0.7483

**Table 12:** RPN proposal recall comparison of different feature-routing variants.

Variant	AR@1000	${AR}_{S}@1000$	${AR}_{M}@1000$	${AR}_{L}@1000$
CBv2 Faster R-CNN baseline	0.993	0.679	0.900	0.996
Direct feature replacement	0.984	0.663	0.874	0.987
Residual routing without identity preservation	0.992	0.642	0.905	0.994
BISRF-Net	0.989	0.705	0.901	0.991

**Table 13:** Computational complexity comparison between the baseline and BISRF-Net.

Model	Input Size	Params	FLOPs	GPU Memory
CBv2 Faster R-CNN	1024 × 1024	69.25 M	0.289 T	876 MB
BISRF-Net	1024 × 1024	83.62 M	0.391 T	967 MB

## Data Availability

The original dataset is available at: https://github.com/serendipitylxd/mmdetection_trout, accessed on 10 May 2026. The processed data and experimental materials generated during this study are available from the corresponding author upon reasonable request.
